# Transparent Perfect Microwave Absorber Employing Asymmetric Resonance Cavity

**DOI:** 10.1002/advs.201901320

**Published:** 2019-08-08

**Authors:** Heyan Wang, Yilei Zhang, Chengang Ji, Cheng Zhang, Dong Liu, Zhong Zhang, Zhengang Lu, Jiubin Tan, L. Jay Guo

**Affiliations:** ^1^ Ultra‐precision Optical & Electronic Instrument Engineering Center Harbin Institute of Technology Harbin 150001 China; ^2^ Key Lab of Ultra‐precision Intelligent Instrumentation (Harbin Institute of Technology) Ministry of Industry and Information Technology Harbin 150080 China; ^3^ Department of Electrical Engineering and Computer Science University of Michigan Ann Arbor MI 48109 USA; ^4^ MIIT Key Laboratory of Thermal Control of Electronic Equipment School of Energy and Power Engineering Nanjing University of Science and Technology Nanjing 210094 China

**Keywords:** graphene, optically transparent, perfect microwave absorption, ultrathin doped silver

## Abstract

The demand for high‐performance absorbers in the microwave frequencies, which can reduce undesirable radiation that interferes with electronic system operation, has attracted increasing interest in recent years. However, most devices implemented so far are opaque, limiting their use in optical applications that require high visible transparency. Here, a scheme is demonstrated for microwave absorbers featuring high transparency in the visible range, near‐unity absorption (≈99.5% absorption at 13.75 GHz with 3.6 GHz effective bandwidth) in the Ku‐band, and hence excellent electromagnetic interference shielding performance (≈26 dB). The device is based on an asymmetric Fabry–Pérot cavity, which incorporates a monolayer graphene and a transparent ultrathin (8 nm) doped silver layer as absorber and reflector, and fused silica as the middle dielectric layer. Guided by derived formulism, this asymmetric cavity is demonstrated with microwaves near‐perfectly and exclusively absorbs in the ultrathin graphene film. The peak absorption frequency of the cavity can be readily tuned by simply changing the thickness of the dielectric spacer. The approach provides a viable solution for a new type of microwave absorber with high visible transmittance, paving the way towards applications in the area of optics.

## 1 Introduction

Microwave absorbers (MAs) are of critical importance in numerous applications, e.g., achieving electromagnetic interference (EMI) shielding,^1^, ^2^, ^3^ enhancing energy harvesting efficiency,^4^ and improving detector sensitivity.^5^, ^6^ In light of the prevalent use of various optoelectronic devices including wearable sensors and smart phones and the associated complex electromagnetic environment, the EMI shielding technology is becoming even more crucial in recent years.^7^, ^8^, ^9^, ^10^, ^11^ Moreover, in many application scenarios, such as windows and displays for aircrafts, aerospace exploration facilities, and optical detecting devices used in medical and electronic safety areas, it is highly desirable that the absorbers can provide high visible transmittance and efficient microwave attenuation simultaneously.^12^, ^13^, ^14^, ^15^

There is a significant effort to explore MAs and methods to improve their absorption performance. For instance, carbon‐based materials including carbon nanotubes, carbon composites, and graphite nanosheets are widely utilized due to their lightweight and efficient microwave absorbing characteristics.^16^, ^17^, ^18^, ^19^, ^20^, ^21^, ^22^ However, they also absorb visible light, which greatly hinder their optical applications. Although atomically thin layer graphene presents extremely low optical loss,^23^ the reported microwave absorption is less than 40% within 2.2–7 GHz.^24^ The implementation of semitransparent MAs have been attempted using other graphene‐based structures, such as graphene/polymers and graphene/silica stacks; however, the overall transparency is inevitably sacrificed, and the microwave absorption has not been greatly improved.^25^, ^26^ Therefore, carbon‐based materials alone are not very good choices for simultaneously achieving high optical transparency and efficient microwave absorption. Metal‐based structures, like ultrathin metal films, metal nanowires, and microscale metal meshes are another option considering their high visible transmittance and strong EMI shielding capabilities.^27^, ^28^, ^29^, ^30^, ^31^ However, these transparent conductors generally produce strong backscattered energy, causing secondary electromagnetic radiation pollution. Recent interest in metamaterials has also led to semitransparent microwave metamaterial absorbers through minimizing reflection by impedance matching.^32^, ^33^, ^34^, ^35^, ^36^, ^37^, ^38^, ^39^, ^40^ Although such structures offer great potential for perfect microwave absorption, their applications are largely limited by the undesired narrow‐band performance.

In this work, we present a general strategy to design the new type of transparent perfect MA based on asymmetric Fabry–Pérot resonant cavity by employing a multilayer structure in the form of graphene‐dielectric‐ultrathin doped silver (GDS). Here the optically transparent graphene and an ultrathin doped silver (Ag) film function as the microwave absorber and reflector respectively, and fused silica as the dielectric spacer. In addition, by tuning the dielectric spacer thickness to achieve critical coupling in the cavity, we show that this device yields near‐unity (≈99.5%) microwave absorption and remains highly transparent (≈74%) in the visible range, and the relative visible transmittance of the cavity can reach ≈93.5%. Furthermore, by employing the simple building block, an ultrathin planar graphene film, we theoretically and experimentally demonstrate that nearly all the microwave is absorbed in the graphene layer by this transparent MA in contrast to other metamaterials and metasurface absorbers where microwave is mainly absorbed in the tailored metallic components (2D arrays of subwavelength elements).

## 2 Results and Discussion

### 2.1 Design and Fabrication of the Transparent Microwave Absorber

**Figure 1:**
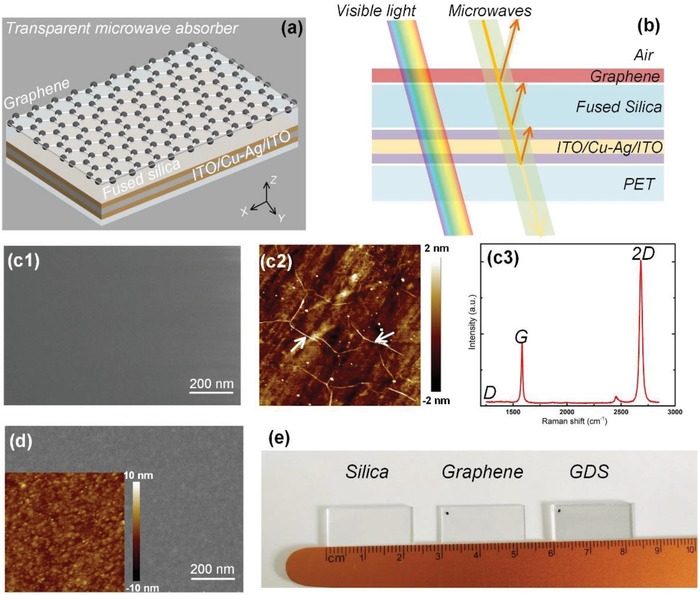
a) Schematic of the transparent MA structure. b) Conceptual diagram illustrating multiple reflection of incident microwaves. c1) SEM and c2) AFM images (500 nm × 500 nm) of CVD‐grown graphene on silica substrate. c3) Raman spectra of the graphene on silica substrate (532 nm laser wavelength). d) SEM and AFM (500 nm × 500 nm) images of ITO/Cu‐doped Ag/ITO on PET substrate. e) Photographs of centimeter‐scale silica, graphene on silica substrate, and the GDS cavity sample, all showing good transparency.

The microwave absorption by a single graphene layer is very limited. However, with its partially microwave absorbing and reflecting properties, graphene can be an excellent candidate as the top absorbing medium in an asymmetric Fabry–Pérot cavity, where one of the mirrors has significantly higher loss than the other. A schematic view of the proposed visibly‐transparent MA based on cavity resonance is depicted in **Figure** **1**a,b. The device simply consists of monolayer graphene, ultrathin Ag and fused silica. Ag is chosen as the back reflection mirror since it is highly conductive to provide strong power reflection in the microwave regime and has the lowest absorption loss among metals in the optical range. Specially, we recently proposed a new approach to achieve ultrathin smooth Ag films by introducing a small amount of additive metal into Ag during the codeposition process (Figure S1, Supporting Information), and employed them as semitransparent electrode in high‐efficiency organic solar cells.^41^, ^42^ In this work, we further investigate ultrathin copper (Cu) doped Ag film as transparent microwave reflective layer in asymmetric cavity configuration for the first time, demonstrating its great potential for high‐performance microwave absorbing devices. The continuous ultrathin (8 nm) and smooth Ag film was obtained by suppressing the Volmer–Weber island growth mode of Ag. The surface morphology of the obtained 8 nm Cu‐doped Ag is shown in Figure S2 (Supporting Information), exhibiting a smooth surface feature with a sub‐nm root‐mean‐square roughness. Furthermore, one strategy to increase optical transmittance of the thin doped Ag film is to employ antireflection coatings, hence, the ultrathin doped Ag layer is sandwiched between two conductive indium tin oxide (ITO) layers in our design. The thickness of ITO layers is optimized to maximize the overall transparency, and the calculated results using transfer matrix method (TMM) are shown in Figure S3 in the Supporting Information. The optimized structure for transparency, ITO (40 nm)/Cu‐doped Ag (8 nm)/ITO (40 nm), further increases the conductance of the stack, providing stronger reflection for the microwave signal.

**Figure 2:**
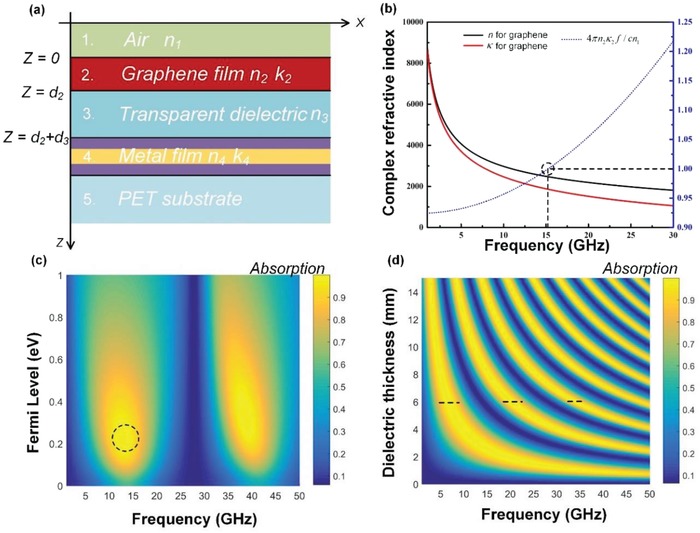
a) Calculation model of the transparent MA consisting of graphene, transparent dielectric, and ultrathin Ag on the PET substrate. b) Complex refractive index (*n* and *k*) of graphene in the microwave region and calculated perfect absorption point using Equation (4). Calculated microwave absorption spectra as a function of c) Fermi level of graphene and d) dielectric thickness from 1 to 50 GHz.

**Figure 3:**
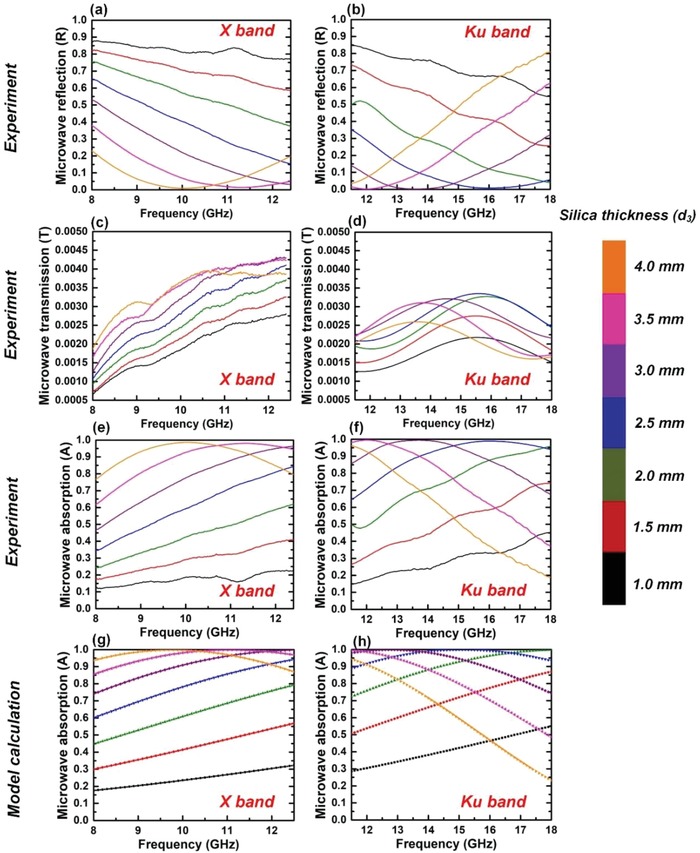
a,b) Mesaured microwave reflection, c,d) transmission, and e,f) absorption spectra for GDS cavities as a function of the silica thickness in the X and Ku bands. g,h) Model calculation of microwave absorption for GDS cavity using TMM (μ_c_ = 0.3 eV, Γ = 20 ps).

The designed GDS cavity can be easily fabricated. The synthesis process of the graphene by the typical chemical vapor deposition (CVD) method is shown in Figure S4 in the Supporting Information. Figure 1c‐(1) and c‐(2) shows the scanning electron microscopy (SEM) and atom force microscope (AFM) images of the graphene film on the silica substrate after transferred from the Cu foil, respectively, and without macroscopic defects. The small white dots in the Figure 1c‐(2) are the polymethyl‐methacrylate (PMMA) residuals after removal process. To verify the quality of the prepared graphene, we measure the Raman spectrum with the laser wavelength of 532 nm as shown in Figure 1c‐(3). It exhibits typical peak features as monolayer graphene, including G and *2*D bands. The *2*D band exhibits a single Lorentzian line shape with a full width at half maximum (FWHM) of ≈26 cm^−1^ located at ≈2686 cm^−1^ and a higher intensity relative to the *G* band. In addition, the intensity of *D* band at ≈1350 cm^−1^ is below the Raman detection limit, which proves the absence of a significant number of defects. Afterwards, the Fermi energy level of graphene is determined by comparing the G and *2*D peak positions we measured, with the characterized correlation from Ref. 50, which is estimated as μ_c_ = ≈0.3 eV.^43^

The doped Ag and ITO layers were deposited on polyethylene terephthalate (PET) substrate by sputtering. SEM and AFM images of the as‐deposited ITO/Cu‐doped Ag/ITO are shown in Figure 1d, all showing the continuous and smooth surface morphologies. Figure 1e shows optical images of silica, graphene film onto the silica, and the ultimate GDS cavity samples. The monolayer graphene film on the silica (in the middle) is highly transparent with little transmittance loss compared with the pure silica (left). The GDS cavity with the size of 2.2 cm × 1.1 cm (right) also shows a favorable transparency.

### 2.2 Theoretical Condition and Experimental Verification for Perfect Microwave Absorption

As shown in **Figure** **2**a, eight parameters (*n*_1_, *n*_2_, *k*_2_, *n*_3_, *n*_4_, *k*_4_, *d*_2_, *d*_3_), including complex refractive index and thickness of interested layers, are involved in designing the transparent MA in this configuration. The metal thickness is fixed at 8 nm to ensure both microwave reflection and optical transparency. It is convenient to describe the graphene's property using its conductivity (σ) since that this quantity can be modeled or measured directly ranging from radio wave to optical frequencies, and the corresponding refractive index (*n*) and extinction coefficients (*k*) can be derived from the conductivity.^44^ For theoretical analysis, the conductivity of the graphene could be calculated from Kubo formula with inter‐band and intraband contributions,^45^ and is estimated using the following expressions

(1) $$\sigma_{\mathrm{intra}}=i\frac{2e^{2}k_{B}T}{\pi \hbar^{2}(\omega +i\Gamma )}\mathrm{In}\left(2\cosh\frac{\mu_{c}}{2k_{B}T}\right)$$

(2) $$\sigma_{\mathrm{inter}}=\frac{e^{2}}{4\hbar }\left[\frac{1}{2}\;+\;\frac{1}{\pi }\arctan\left(\frac{\hbar \omega -2\mu_{c}}{2k_{B}T}\right)-\frac{i}{2\pi }\mathrm{In}\frac{{\left(\hbar \omega \;+\;2\mu_{c}\right)}^{\;2}}{{\left(\hbar \omega -2\mu_{c}\right)}^{2}\;+\;{\left(2k_{B}T\right)}^{\;2}}\right]$$

where *e* is the electron charge, *k*_B_ is the Boltzmann constant, *T* is the temperature, ℏ is the reduced Planck constant, ω is the frequency of the incident electromagnetic waves, and Γ is the relaxation time which is assumed to be independent of energy. It is worth nothing that graphene's conductivity strongly depends on the value of its Fermi energy level μ_c_ which can be controlled by chemical doping or electrostatic gating.^46^, ^47^ Specifically, atomically thin graphene is sensitive to foreign atoms or molecules absorbed on the surface and has a pronounced ambipolar electric field effect, indicating that the changes in the position of the Fermi level can be induced by chemical doping or changing gate voltages, thus changing the conductivity.^48^, ^49^ On the other hand, the ITO/Cu‐doped Ag/ITO stacks (microwave reflection mirror) can be modeled as an effective medium layer with high electrical conductivity, as the stack is ultrathin compared with the microwave wavelength and most of the contribution comes from the most conductive Ag layer.

We consider the theoretical condition for perfect microwave absorption in the planar graphene layer by destructive interference through multiple reflections within the GDS cavity. As graphene is the only lossy medium and there is no restriction placed on the metallic substrate in the GDS cavity, so even metal approaching the perfect electric conductor limit can be used. For simplicity, we assume that all the power is consumed by the graphene layer and the bottom reflective layer can provide 100% reflectivity. Here, we adopt an analytical method for the absorber structure studied in the optical regime,^50^ and the absorption rate per unit length normalized to the incident microwaves could be expressed as

(3) $$A_{\mathrm{pul}}(z,f)=\frac{4\pi n_{2}\;\kappa_{2}f}{cn_{1}}\frac{{\left|E(z)\right|}^{\;2}}{{\left|E_{0}\right|}^{\;2}}$$

where *E*(*z*) is the electric field in the ultrathin graphene film, *E*_0_ is the incident electric field, *c* is the light speed in the vacuum, and *f* is the frequency of the incident electromagnetic wave. Therefore, the total absorption in the graphene film is

(4) $$A_{\mathrm{total}}(f)\;=\;\frac{4\;\pi n_{2\;}\kappa_{\;2}f}{cn_{\;1}}\frac{\int_{0}^{d_{\;2}}{\left|E\;(z)\right|}^{\;2}dz}{{\left|E_{0}\right|}^{\;2}}$$

Since the thickness of monolayer graphene (*d*_2_ ≈ 0.34 nm) is much smaller than the wavelength of the incident microwave (*d*_2_ ≪ λ), a sheet of graphene layer minimally changes the electric field profile inside the cavity, and the electric field can maintain its intensity within the monolayer absorber layer, which is also validated by calculating the electric field distribution within the graphene layer at different frequencies (Figure S5, Supporting Information). In view of the zero reflection in the critical coupling condition, we can assume that

(5) $$E(z)=E(z=0)=E_{0},\;0\leq z\leq d_{\;2}$$

Substituting *E*(*z*) = *E*_0_ into Equation (4) and setting *A*_total_ = 100%, the sufficient and necessary condition for perfect microwave absorption is obtained

(6) $$\frac{4\pi n_{2}\;\kappa_{2}d_{2}f}{cn_{1}}=1$$

The dotted line in the Figure 2b plots the calculated values of the 4*πn*_2_κ_2_ *d*_2_ *f*/*cn*_1_ for normal incidence, revealing that perfect absorption at around 15 GHz can be achieved in the GDS cavity by the graphene film under the condition of μ_c_ = 0.3 eV and Γ = 20 ps.

Furthermore, by employing TMM, we can calculate the reflection (*R*) and absorption (*A* = 1−*R*) of the GDS cavity at interested frequencies. Due to the symmetry of the cavity in the *X*–*Y* plane, the device should be independent of polarization at normal incidence. In the following numerical calculations, we only take TE‐polarized incident microwaves into consideration. Figure 2c gives the calculated absorption map as a function of different graphene Fermi levels and frequencies at the dielectric thickness *d*_3_ of 3 mm. It illustrates that the near‐unity absorption of the GDS cavity can be achieved with different Fermi levels in the Region A (black dotted circle), which also shows that the conditions for achieving strong absorption are not very narrow. In addition, the ideal perfect absorption is attained with μ_c_ = ≈0.3 eV at near 15 GHz, which is consistent with the predicted result based on Equation (6). Figure 2d shows the calculated absorption by varying the dielectric thickness (ranging from 0 to 15 mm) at different excitation frequencies. As shown in Figure 2d, the absorption peaks occurs periodically with the increase of frequencies at a fixed dielectric thickness, as well as increasing dielectric thickness at a particular frequency. Specifically, when the dielectric thickness *d*_3_ varies from 1 to 15 mm, the absorption peak shifts continuously from ≈40 to ≈2.5 GHz.

The relationship between dielectric thickness (*d*_3_) and resonant frequency (*f*_0_) can be understood by investigating the condition of partial reflected wave for the destructive interference in the Fabry–Pérot configuration, which can be roughly estimated as *f*_0_ = *c*/(4*n*_3_ *d*_3_). However, due to the strong loss in the graphene layer, the reflection phase of partial reflected wave at the air–graphene interface is not equal to π, resulting in a slight resonant peak shifts under the destructive interference condition. Meanwhile, several higher‐order modes start to appear with thicker cavities and higher frequencies, forming several bands on the absorption map. Hence, it is obvious that the peak absorption frequencies can be readily tuned by changing the dielectric thickness of the cavity. Note that at a fixed dielectric thickness (e.g., *d*_3_ = 6 mm), the bandwidth of lower‐order resonances is slightly broader than that of higher‐order modes (black dashed lines in the Figure 2d), which increases the operating bandwidth and provides better tolerance to experimental verification.

To confirm the microwave absorption performance of the GDS cavity, the microwave transmission (*T*) and reflection (*R*) are measured at normal incidence using waveguide configuration, and then used to calculate absorption (*A*), which is defined as *A* = 1 − *T* − *R*, where *R* = |*S*_11_|^2^ and *T* = |*S*_21_|^2^ are obtained from the measured *S*‐parameters. First, by analyzing the microwave responses of the GDS cavity layers individually, we checked the respective role of each layer. The microwave transmission and reflection of the monolayer graphene on silica substrate and the ITO/Cu‐doped Ag/ITO on PET substrate in the Ku bands are plotted in Figure S6 in the Supporting Information. As predicted, the monolayer graphene is partially reflective and absorptive to the microwaves with averaged ≈25% reflection and ≈45% transmission. On the other hand, besides the high visible transmittance, the ITO/Cu‐doped Ag/ITO layer supports a broadband high microwave reflection, corresponding to only ≈0.3% transmission, which is nearly perfect as a microwave reflection mirror in the cavity.

Then, we examined the microwave absorption performance of the GDS cavities. The measured microwave reflection and transmission for the GDS cavities with different dielectric thicknesses are shown in **Figure** **3**a–d, through which the experimental absorption (Figure 3e,f) can be retrieved. As shown in Figure 3c,d, GDS cavity exhibits a strong EMI shielding capability in the entire X and Ku band due to the back doped Ag mirror, which permits an ultralow microwave transmission through the cavity with EMI shielding effectiveness (SE) of ≈26 dB (*T* ≈ 0.25%) at different frequencies.^51^ From model calculation using TMM, the absorption spectra in the X and Ku band of the GDS cavity are plotted in Figure 3g,h, with silica thickness *d*_3_ ranging from 1 to 4 mm. The overall shape, trend and position of the experimental features in Figure 3e,f are consistent well with the calculated results.

In addition, with the increase of the dielectric thickness, the location of the absorption peaks gradually red‐shifted to the low frequencies and was able to be tuned across the measured spectrum from 8 to 18 GHz. As characterized by the purple curves (*d*_3_ = 3 mm) in the Figure 3e,f, the measured absorption reaches a maximum of 99.5% at 13.75 GHz, showing that the near‐unity absorption is achieved. At the same time in the case of *d*_3_ = 3 mm, broadband absorption is obtained with the measured absorption of >50% from 8 to 18 GHz, covering the entire X and Ku bands, while all maintaining ultralow microwave transmission through the cavities. In addition, the effective bandwidth is defined as the width of the band with microwave absorption intensity of over 90%. For the GDS cavity at *d*_3_ = 3 mm, the measured effective bandwidth is 3.6 GHz, from 12 to 15.6 GHz in Figure 3f. For the other cases where *d*_3_ = 2.5, 3.5, and 4 mm, the maximal absorption values all exceed 98% at their resonant frequencies, showing excellent microwave absorption performance of the GDS cavities.

### 2.3 Asymmetric Fabry–Pérot Cavity

**Figure 4:**
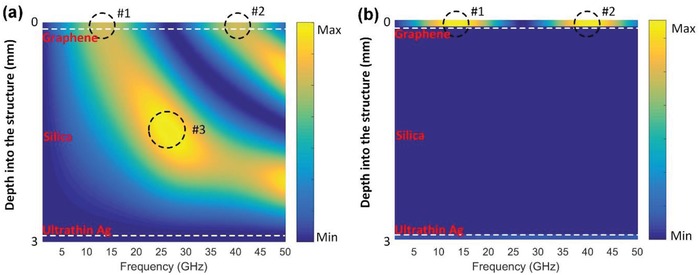
a) Calculated electric field distribution and b) calculated absorbed power distribution within the whole GDS cavity (top graphene layer and bottom Ag layer are separated by the silica) at concerned frequencies.

In order to get a better understanding of the extraordinary microwave absorption by an atomically thin graphene layer, numerical calculations using TMM of the electromagnetic responses,^52^, ^53^ including electric field and absorbed power distributions within the whole cavity (*d*_3_ = 3 mm) at interested wavelengths, are presented in **Figure** **4**. It is noteworthy that the resonance modes in the top graphene layer appear at ≈13 GHz (#1) and ≈40 GHz (#2), as shown in Figure 4a, which matches well with the perfect absorption frequencies at the dielectric thickness of 3 mm in Figure 2d. The enhanced electric field distribution at resonances further unveils the reason for strong absorption in the graphene layer of the cavity as the power absorption is directly proportional to the electric field intensity. The electric field intensity at 25 GHz (#3) is the highest in the GDS cavity at this thickness, but it locates at the middle of the silica layer having negligible absorption in the microwave range. By calculating the absorbed power distribution as a function of frequencies at the same GDS cavity thickness (In Figure 4b), we find the highest absorption positions (#1 and #2) corresponding well to the intensive field regions in Figure 4a. It is obvious that nearly all the microwave power is absorbed by the top graphene layer, which supports the perfect absorption condition expressed in Equation (6). As for the bottom Ag layer, even though the electric field is extremely weak, there is still little power absorbed by the Ag due to its large attenuation coefficient in the microwave range. Note that the thickness of each layers are not drawn to scale in Figure 4, in order to better illustrate the intensive field and high absorption regions in the structure. In addition, recent development of absorbing materials or structures based on graphene with broad effective bandwidth is imperative for adapting to complex electromagnetic environment.^54^, ^55^, ^56^, ^57^ As the microwave power is exclusively absorbed in the graphene layer in this GDS cavity, the effective way to improve absorption bandwidth is to add more graphene layers in the cavity separated by the silica on top of ultrathin doped Ag, which can introduce multiple resonances in different graphene layers at different frequencies, and the overlap of these resonances enables broadband absorption. Furthermore, the thickness of each silica between different graphene layers can be adjusted to control the resonant positions at the designed frequencies.

In simple physics, the GDS cavity resembles an asymmetric Fabry–Pérot (FP) cavity comprising of a lossless dielectric core with a partially reflective top layer and back reflector. At resonances, microwave beams reflected from the air/graphene and the silica/metal interfaces form destructive interference, resulting in zero reflection, which accounts for the period absorption peaks in the Figure 2d at a fixed dielectric thickness. Nevertheless, it is essentially distinctive from the conventional FP cavity, especially by incorporating lossy graphene layer in such an asymmetric FP cavity, the structure can yield extraordinary power absorption.

### 2.4 Optical Performance

**Figure 5:**
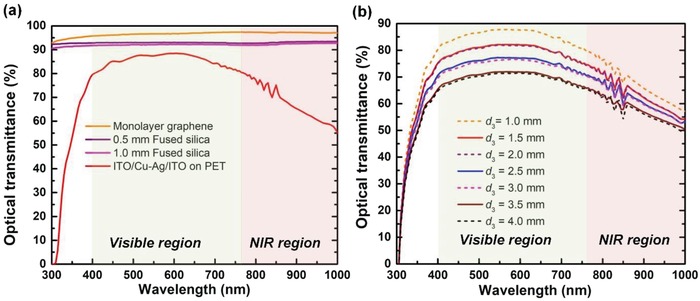
Optical transmittance spectra of a) 0.5 and 1 mm silica, ITO/Cu‐doped Ag/ITO on PET substrate and monolayer graphene and b) GDS cavities with different silica thickness.

The thickness of such GDS cavity is on the order of several millimeters, which is much larger than the visible wavelengths, so it is hard for the absorbing layer and reflective layer to form an optical cavity in this case due to the incoherent situation. Therefore, the optical transmittance of the GDS cavity can be improved by enhancing the transparency of individual layers or reducing dielectric layers. To characterize the optical property of the GDS cavity, we measured transmittance spectra in the range of 300–1000 nm of the individual layers and GDS cavities with different dielectric thicknesses, as shown in the **Figure** **5**. The as‐prepared graphene and silica substrates (0.5 and 1 mm) shows nearly flat transmission spectra in the visible and near infrared regions, with 96.7%, 93%, and 92% visible transmittance at 550 nm respectively. The averaged transmittance of the ITO/Cu‐doped Ag/ITO on PET substrate is 88.1% in the visible band, and the improved transparency than the thin Ag film alone is due to ITO acting as antireflection coatings on the Cu‐doped Ag layer. Moreover, at longer wavelengths, it approaches that of a perfect conductor and its reflection increases towards the infrared regime. As a consequence, film transmittance gradually drops as depicted in the red curve in the Figure 5a, but still remains a relatively high value (e.g., 65% at 900 nm).

Similar to the ITO/Cu‐doped Ag/ITO layer, there is a downward trend of transmittance spectra for GDS cavities with increasing wavelengths at different dielectric thicknesses in the near infrared range. On the other hand, as depicted in the Figure 5b, the transmittance drops when silica thickness increases, which is attributed to the reflection loss at interfaces and possible absorption loss in the substrates. In our experiments, each different dielectric thickness for the cavity (1–4 mm) is a simple stacking of 0.5 and 1 mm silica substrates, where small air gaps between each layer is anticipated. As a result, the stepped optical losses in the transmission spectra (e.g., from 1.0 to 1.5 mm/from 2.0 to 2.5 mm/from 3.0 to 3.5 mm) is mainly caused by the reflection loss on the air/silica interface in the cavities. For the case where *d*_3_ = 3 mm, we have achieved the near‐unity absorption in the microwave range, and an averaged visible transmittance of 74% (relative to PET substrate) can still be maintained. It is important to note that the transparency of the GDS cavity can be improved by using one single dielectric spacer instead of employing multilayer configuration at the designed thickness, and the visible transmittance of the GDS cavity will be enhanced by eliminating the interface reflection loss up to nearly 87% (like *d*_3_ = 1 mm case in Figure 5d).

**Table** **1** shows the optical and microwave absorption performance reported for different transparent microwave absorbers. The GDS cavity prepared in this study (*d*_3_ = 3 mm) could achieve an EMI SE of ≈26 dB, a microwave absorption of ≈99.5% with visible transparency of ≈74% (and can be improved up to >85% by using single layer of silica). With a lower microwave absorption, the GDS cavity with 1‐mm‐thick dielectric spacer shows a visible transmittance of ≈87%. All these values match or outperform most of the reported results shown in the table.

**Table 1:** Microwave and optical properties of different transparent microwave absorbers

Microwave absorber	SE [dB]	Microwave absorption	Transparency	Relative transmittance	Ref.
Monolayer graphene	2.27	<40%	–	–	Hong et al.^24^
Single‐PEI/RGO	3.09	13.79%	73% at 700 nm	–	Kim et al.^25^
Double‐PEI/RGO	6.37	48.26%	62% at 700 nm	–	Kim et al.^25^
Eight‐layer graphene/PET	19.14	95.82%	–	80.5% at 500 nm	Ma et al.^58^
Four‐layer graphene/PET	10.01	86.69%	–	89.6% at 500 nm	Ma et al.^58^
Graphene/PMMA	–	25%	97.8%	–	Batrakov et al.^26^
Graphene/quartz (four layers)	–	90%	85%	–	Wu et al.^26^
Bow‐tie metamaterials	–	90%	62%	–	Jang et al.^38^
Windmill‐shaped metamaterials	–	90%	77%	–	Zhang et al.^59^
GDS cavity (*d*_3_ = 3 mm)	26	99.5%	74%–87%	93.5% at 550 nm	This work

## 3 Conclusions

In summary, we have experimentally demonstrated a transparent and near‐unity absorption microwave absorber consisting of graphene, transparent spacer and ultrathin doped Ag based on asymmetric FP cavity. The experimental results are consistent with the theoretical calculations. The physical origin can be explained by the asymmetric FP cavity model. Experimental results show a maximal absorption up to ≈99.5% at 13.75 GHz, an EMI shielding effectiveness of 26 dB with an averaged visible transmittance of 74% (limited by the light reflection from the air/silica interface used in the current experiment). In addition, the GDS cavity is easy to be tuned either by chemical doping or electrostatic gating for the graphene layer or simply altering the cavity thickness. The devised configuration and physical insight opens up a new pathway for transparent microwave absorbing materials and for developing novel microwave optical components.

## 4 Experimental Section

*Graphene Synthesis*: The graphene films were grown on 25 µm thick Cu foils (Alfa Aesar, 99.8% purity) using CVD method at the center of a tube furnace. The foils were subsequently heated to 1000 °C under H_2_ at 60 Pa for half an hour. Then, the carbon source (CH_4_) was introduced into the quartz tube for 2 h. After following gas mixtures, we rapidly cooled down the samples to the room temperature (25 °C). The fast cooling rate is critical in suppressing the multiple layers formation of graphene.

*Graphene Transfer*: As‐grown graphene on the Cu foils was transferred by spin‐coating 5 µm thick PMMA on the graphene. After PMMA dried, the sample was immersed into the Marble's etchant (HCl: H_2_SO_4_: CuSO_4_ = 50 mL: 50 mL: 10 g) solvent to etch the Cu foil. After removal, the graphene sample was washed in distilled water and transferred to the 1 mm silica substrate to be used.

*Film Deposition*: Cu‐doped Ag films were fabricated by co‐sputtering of Cu and Ag. The concentration of Cu and Ag was controlled by adjusting the individual sputtering power of the Cu and Ag targets. The calibrated deposition rates for the Cu‐doped Ag film employed in this study are 1.109 and 0.019 nm s^−1^ for Ag and Cu, respectively, which leads to ≈2% atomic concentration of Cu in the obtained film. The film thickness was controlled by the deposition time. The chamber base pressure was pumped down to about 0.13 mPa before film deposition. During deposition, the Ar gas pressure was 0.6 Pa and the substrate holder was rotated at the speed of 10 rpm.

*Characterization and Measurements*: ITO/Cu‐doped Ag/ITO film thickness of each layer was calculated by the calibrated deposition rates and confirmed by the spectroscopic ellipsometry measurement (J. A. Woollam M‐2000). The morphology of the graphene and ITO/Cu‐doped Ag/ITO films were investigated by SEM (FEI HELIOS Nanolab 600i) and tapping mode AFM (Bruker Dimension FastScan) on fused silica and PET substrates, respectively. The optical transmittance of the films and GDS cavities were measured by a UV‐vis–Infrared spectrophotometer (PerkinElmer Lambda 950) ranging from 300 to 1000 nm with normal incident radiation. The microwave transmission and reflection of the samples was calculated from the measured *S*‐parameters (*S*_11_ and *S*_21_) using waveguide methods in the X and Ku bands. Schematic illustration and microwave measurement setup and are shown in the Figure S7 in the Supporting Information.

## Conflict of Interest

The authors declare no conflict of interest.

## Supporting information

Supplementary
