# Supplementary material for: Transparent Perfect Microwave Absorber Employing Asymmetric Resonance Cavity
Source: Adv Sci (Weinh). 2019 Aug 8;6(19):1901320. doi: 10.1002/advs.201901320 (PMC6774038; doi:10.1002/advs.201901320)
Supplement: Supplementary file 1 — Supplementary [file ADVS-6-1901320-s001.pdf]

Copyright WILEY-VCH Verlag GmbH & Co. KGaA, 69469 Weinheim, Germany, 2019.

ADVANCED SCIENCE
Open Access

Supporting Information

for *Adv. Sci.,* DOI: 10.1002/advs.201901320

Transparent Perfect Microwave Absorber Employing Asymmetric Resonance Cavity

Heyan Wang, Yilei Zhang, Chengang Ji, Cheng Zhang, Dong Liu, Zhong Zhang, Zhengang Lu, Jiubin Tan, and L. Jay Guo**

Copyright WILEY-VCH Verlag GmbH & Co. KGaA, 69469 Weinheim, Germany, 2019.

Supporting Information

Transparent Perfect Microwave Absorber Employing Asymmetric Resonance Cavity

Heyan Wang, Yilei Zhang, Chengang Ji, Cheng Zhang, Dong Liu, Zhong Zhang, Zhengang Lu, Jiubin Tan and L. Jay Guo**

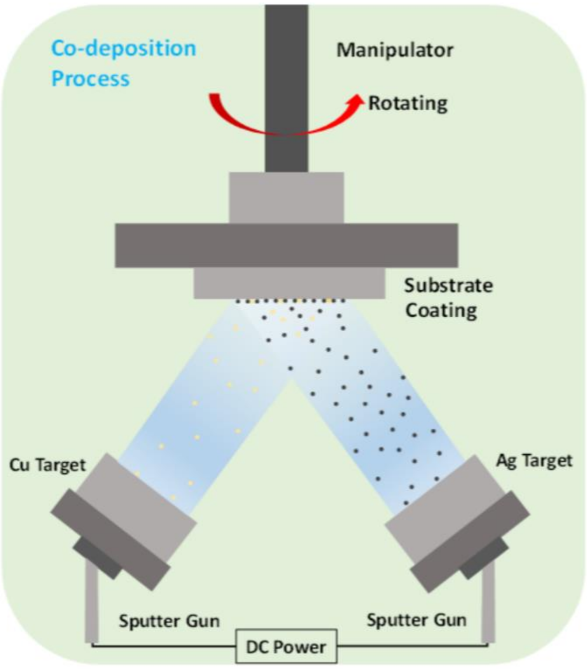

Figure S1. Set-up of co-deposition of Cu and Ag.

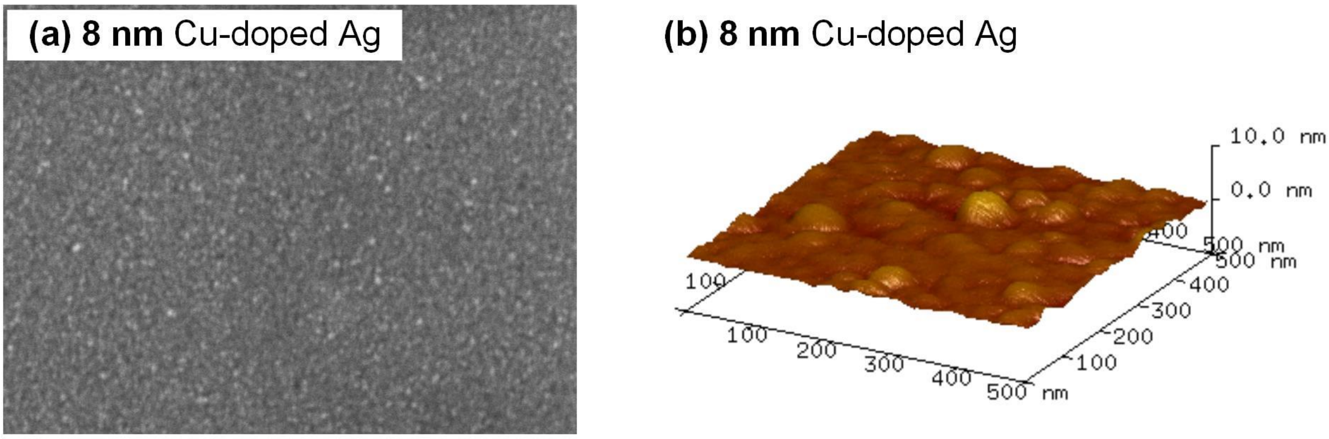

Figure S2. Surface morphology of 8 nm Cu-doped Ag film. a) SEM image. b) AFM image.

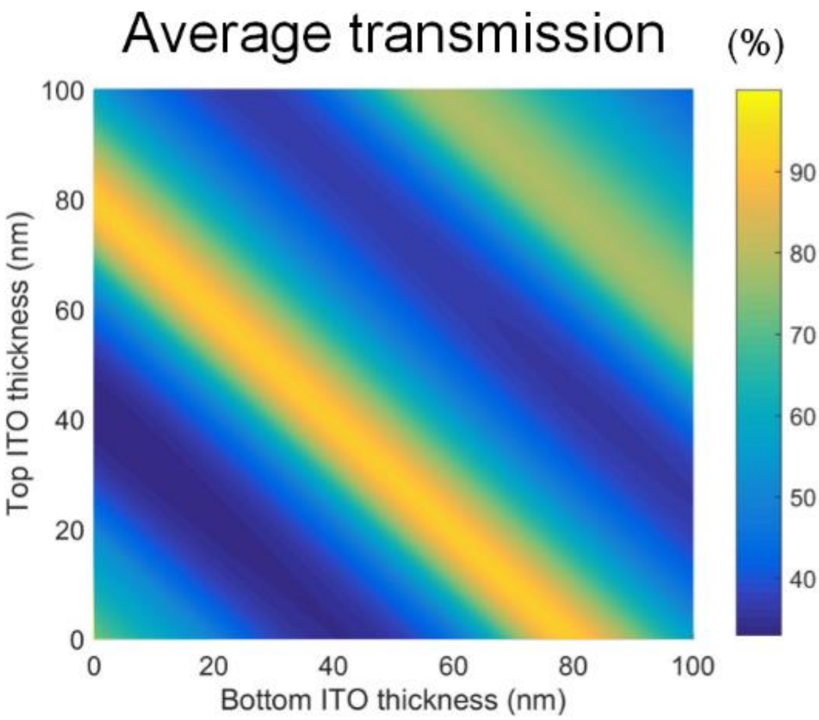

Figure S3. Calculated average visible transmittance for various thickness of the top and bottom ITO layers on 8-nm Ag.

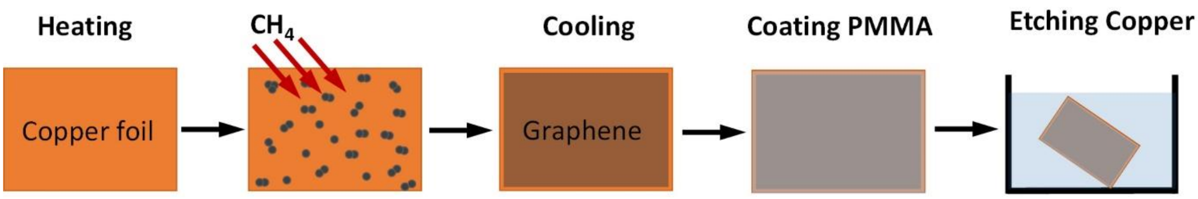

Figure S4. Synthesis and transfer process for graphene films.

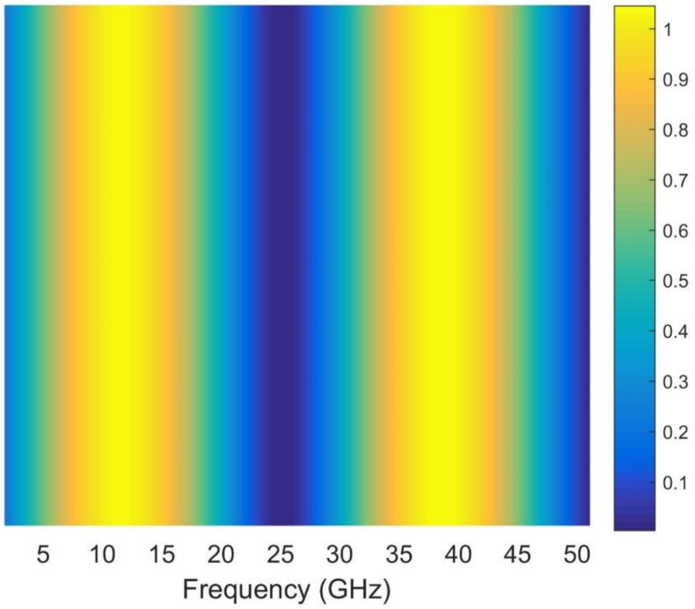

Figure S5. Calculated electric field distribution within the graphene in the GDS cavity layer at concerned frequencies. It shows that at resonant frequencies the electric field intensity is close to 1 (same to the incident field), and the electric field is stable at one particular frequency within the graphene

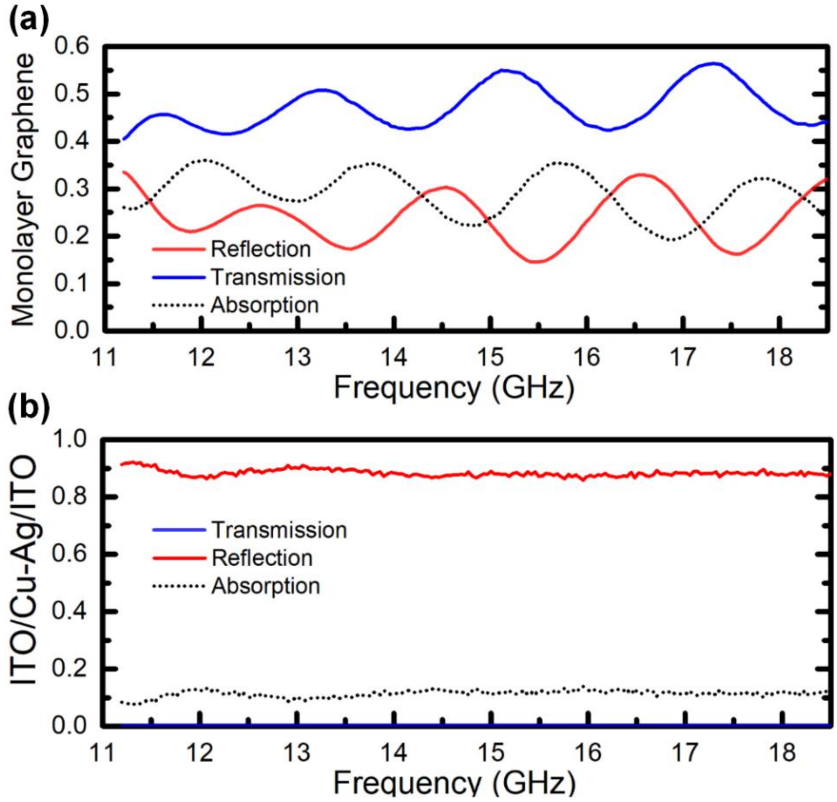

Figure S6. Microwave transmission (T), reflection (R) and absorption (A) for a) monolayer graphene on 1 mm thick silica substrate and b) ITO/Cu-doped Ag/ITO on 50 μm thick PET substrate.

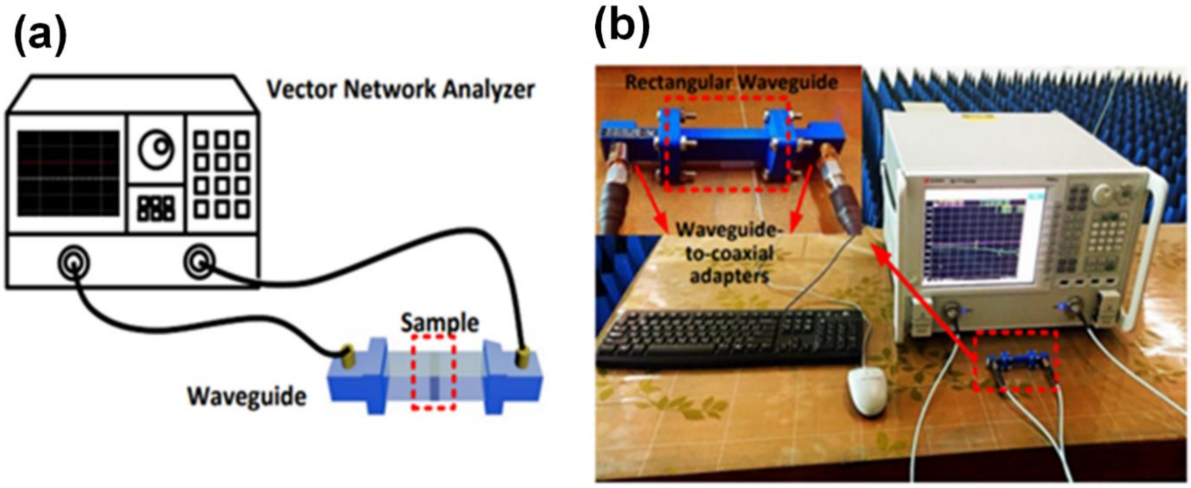

Figure S7. a) Schematic illustration and b) photograph of the measurement setup for the EMI shielding. Inset in the b) shows the rectangular waveguide of the Ku band.
